# Predictive value of dynamic renal resistive index (drin) for renal outcome in type 2 diabetes and essential hypertension: a prospective study

**DOI:** 10.1186/s12933-015-0227-y

**Published:** 2015-05-22

**Authors:** R. M. Bruno, A. Salvati, M. Barzacchi, K. Raimo, S. Taddei, L. Ghiadoni, A. Solini

**Affiliations:** Institute of Clinical Physiology – CNR, Via Moruzzi 1, 56125 Pisa, Italy; Department of Clinical and Experimental Medicine, University of Pisa, Via Roma 67, 56124 Pisa, Italy

**Keywords:** Renal resistive index, Hypertension, Type 2 diabetes, Arterial stiffness, Renal outcome, Prospective study, Microalbuminuria

## Abstract

**Background:**

Hypertension (EH) and type 2 diabetes (T2DM) are major causes of chronic kidney disease (CKD) and identification of predictors of CKD onset is advisable. We aimed to assess whether dynamic renal resistive index (DRIN), as well as other markers of systemic vascular damage, are able to predict albuminuria onset and estimated glomerular filtration rate (eGFR) decline in patients with T2DM or EH.

**Methods:**

In this prospective observational cohort study, 27 T2DM and 43 EH patients, free of CKD at baseline, were followed-up for 4.1 ± 0.6 years. Resistive Index (RI), endothelium-dependent (FMD) and independent vasodilation in the brachial artery (after glyceryl trinitrate – GTN - 25 μg s.l.), carotid-femoral Pulse Wave Velocity (PWV), Augmentation Index (AIx), DRIN (%RI change after GTN 25 μg s.l.) were evaluated.

**Results:**

Patients developing microalbuminuria were older, more frequently T2DM, with higher UACR at baseline, and showed higher DRIN (−2.8 ± 6.7 vs −10.6 ± 6.4 %, p = 0.01) and PWV (9.9 ± 1.3 vs 7.9 ± 1.5 m/s, p = 0.004) at baseline. The best predictors of microalbuminuria onset were DRIN > −5.16 % in T2DM (sensitivity 0.83, specificity 0.80) and PWV > 8.6 m/s in EH (sensitivity 0.96, specificity 1.00). Individuals whose eGFR declined (n = 27) had higher eGFR at baseline, but similar vascular characteristics; however in EH showing eGFR decline, baseline DRIN and PWV were higher. PWV showed a steeper progression during follow-up in patients developing albuminuria (Visit-outcome interaction: p = 0.01), while DRIN was early compromised but no further impaired (Visit-outcome interaction: p = 0.04).

**Conclusions:**

PWV and DRIN are able to predict microalbuminuria onset in newly diagnosed EH and T2DM. DRIN is early compromised in T2DM patients developing microalbuminuria.

## Background

Chronic kidney disease (CKD) is a major cause of cardiovascular morbidity and mortality, with hypertension and type 2 diabetes (T2DM) representing the most relevant risk factors for its development in western countries [1]. Both increased albumin excretion and reduction in glomerular filtration rate are associated with increased incidence of all-cause and cardiovascular mortality in the general population and in T2DM individuals [2–4]. Thus, great attention has been paid to look for early biomarkers able to predict CKD onset [5].

On regard of large arteries, endothelial dysfunction and arterial stiffness are early vascular pathological changes that might lead to end-organ dysfunction [6–8] and are able to predict cardiovascular events in high-risk patients such as those with diabetes [9, 10]. However, the association between arterial stiffness and decline in renal function in longitudinal studies is controversial. Aortic pulse wave velocity (PWV), the gold standard technique for measuring aortic stiffness, was independently associated with a further loss of renal function in CKD patients from the ACADEMIC Study [11], but not in the general population cohort of the Framingham Heart Study [12]. Furthermore, in a cohort of 461 T2DM patients followed up to 5 years, carotid-femoral PWV was an independent predictor of microalbuminuria (MA) onset and correlated with annual change in estimated glomerular filtration rate (eGFR) [13].

Renal resistive index (RI) is a duplex ultrasound-derived parameter, related to renal arteriolosclerosis [14], and it may represent an integrated index of arterial compliance, pulsatility and downstream microvascular impedance [15, 16]. Among its applications in the clinical management of a number of renal conditions, high RI has a negative prognostic value in T2DM patients in terms of progression of renal disease [17]. However, some limitations in its use and interpretation should be acknowledged, and its clinical significance has been recently questioned [18, 19].

A few years ago, we identified a new ultrasound-based biomarker, which we called Dynamic renal Resistive INdex (DRIN), consisting in the change in RI after sublingual nitrate administration and conceivably representing an index of renal vasodilating capacity. DRIN is altered in newly diagnosed, untreated hypertensive patients, and further compromised in T2DM patients without renal damage [20]. DRIN, but not RI, correlated with parameters of vascular function and arterial stiffness, beyond the effect of classical cardiovascular risk factors. DRIN was related with metabolic and blood pressure control in T2DM patients, and with arterial stiffness and wave reflections in hypertensive individuals, suggesting different routes of renal vascular damage accrual in the early stages of the two conditions [20].

The aim of this pilot study has been to evaluate prospectively whether DRIN, as well as other markers of systemic vascular damage, is able to predict microalbuminuria onset or eGFR decline in hypertensive and T2DM patients.

## Methods

### Patients

The original cohort [20] included 32 newly diagnosed (<3 months), treatment-naive T2DM patients, defined according to the American Diabetes Association criteria and 49 never treated essential hypertensive patients (EH). Modality of enrollment and inclusion/exclusion criteria have been previously described [20]. Briefly, inclusion criteria were: age between 40 and 70 years; written informed consent; diagnosis of essential hypertension (for the EH group) or type 2 diabetes (for the T2DM group) within the previous 6 months, according to current guidelines, absence of any micro or macrovascular complications, normal albumin excretion rate in the previous six months, no previous or current treatment with antihypertensive or antidiabetic medications. The protocol was approved by the local ethical committee and all patients gave a written consent.

### Experimental protocol: Visit 0

At Visit 0 the patients underwent a comprehensive evaluation, including blood and urine samples collection, baseline and dynamic renal RI, flow-mediated dilation of the brachial artery (FMD) and carotid-femoral PWV and Aix. A detailed description of the methods is present in the published article regarding the cross-sectional evaluation at Visit 0 [20].

#### Baseline and dynamic renal resistive index

The duplex ultrasound intraparenchimal renal scan (MyLab 25, ESAOTE Florence, Italy) was performed by one trained operator using a high resolution multifrequency Convex probe (2.5 - 4.5 MHz). Three velocimetric measurements of the interlobar renal arteries were obtained at baseline and five minutes after sublingual administration of glyceryl trinitrate 25 μg (GTN), at the end of the brachial artery evaluation (see below), so that a single GTN administration was given to each patient. RI was calculated as: (systolic peak velocity – end diastolic velocity)/systolic peak velocity. DRIN (%) was calculated as: (postGTN RI - baseline RI) ×100/baseline RI [20, 21].

#### Endothelium-dependent and -independent vasodilation in the brachial artery

Endothelium-dependent response was assessed by flow-mediated dilation (FMD), as previously described [22]. Briefly, a pediatric cuff was positioned around the right forearm and the right brachial artery was located and scanned using a 10 MHz linear array transducer (MyLab 25, ESAOTE Florence, Italy). After 1-min baseline recording, the cuff was inflated for 5 min at 300 mmHg and then deflated to induce reactive hyperemia. Endothelium-independent vasodilation was obtained by the sublingual administration of 25 μg GTN [22]. Brachial artery diameter and flow velocity were continuously monitored by computerized edge detection system (Cardiovascular Suite; Quipu srl, Italy) [23]. FMD and response to GTN were calculated as the maximal percent increase in diameter above baseline.

#### Arterial tonometry

Arterial tonometry (SphygmoCor, AtCor Medical, Sidney, Australia) was performed according to the international recommendations [6]. Central blood pressure was derived from radial pressure waveform by means of a validated transfer function and averaged on three measurements. Augmented pressure was calculated as the difference between the second and the first systolic peak, and augmentation index (AIx) was calculated as the ratio between augmented pressure and pulse pressure (PP) and normalized at a heart rate of 75 bpm. Time to reflection (TR) was defined as the total travel time of the pulse-wave to the periphery and its return. Carotid-femoral pulse wave velocity (PWV) was calculated as the ratio of the surface distance between the two recording sites (subtracted distance) and wave transit time. In 57 out of 70 patients radial arterial tonometry and brachial BP were taken after GTN administration, during RI measurements, in order to collect central BP and wave reflection variables.

### Experimental protocol: Visit 1

27 out of the 32 T2DM and 43 of the 49 EH forming the original cohort agreed to participate in the follow-up and formed the population of the present prospective, observational, pilot study.

Visit 1 took place after a mean period of 4.1 ± 0.6 years from Visit 0 (range 3.4-5.1 years). Patients were asked to refer to the Hypertension Outpatient Clinic after an overnight fasting. A blood sample was drawn from an antecubital vein and fasting glucose, HbA_1c_, serum creatinine, total and HDL cholesterol and triglycerides were measured by standard techniques. eGFR was estimated using the CKD-EPI formula [24]. Urinary albumin excretion was evaluated as urine albumin to creatinine ratio (UACR) in two consecutive spot morning urine samples; when micro-macroalbuminuria was detected, the result was confirmed in a 24-h urine collection within the following four weeks. Plasma nitrotyrosine was analyzed by chemiluminescence (Nitrotyrosine Assay Kit, Merck Millipore; Merck KGaA, Darmstadt, Germany), according to the manufacturer’s instructions, in blood samples collected during Visit 1 and in frozen samples collected during Visit 0, since the previously used Nitrotyrosine ELISA Test Kit (Cell Sciences, Canton, MA, USA) [20] was no longer available. A detailed pharmacological and clinical history was collected, and a complete clinical examination was performed. Clinic BP was then measured (mean of at least two measurements in 5 min in the supine position by an automatic sphygmomanometer – OMRON M4). At Visit 1 patients underwent also baseline and dynamic renal resistive index and arterial tonometry, performed by the same modality of Visit 0.

The following renal clinical outcomes were considered:

- Microalbuminuria onset: defined as UACR ≥ 30 mg/g present at Visit 1 and confirmed within 4 weeks;
- eGFR decline: defined when the slope of eGFR was < −1 ml/min1.73 m^2^/year along the follow-up, assuming the physiologic age-related GFR decline as approximately 1 ml/min/year. eGFR slope was defined as the regression coefficient between eGFR and time in units of ml/min per 1.73 m^2^/year, plotting at least three eGFR measurements during the follow-up period [25].

### Statistical analysis

Statistical analysis was performed using NCSS 8 (NCSS, Kaysville, Utah; USA). The results were expressed as mean ± SD for normally distributed variables and as median (25 %-75 %) for not-normally distributed variables. Differences between groups (T2DM vs EH; MA vs No MA; eGFR decline vs No GFR decline) were analyzed using t-test for normally distributed variables, and Wilcoxon Rank Sum test for not normally distributed variables; categorical variables were analyzed by χ^2^ test. Analysis of covariance (ANCOVA) was used to analyze differences in vascular variables between groups with favorable or unfavorable outcomes, considering age and mean BP as covariates. The behavior of variables over time in patients developing or not an unfavorable outcome was assessed by repeated measures ANOVA, testing two-factor (visit-outcome) interaction and using Bonferroni test to analyze multiple comparisons. Receiver operating characteristic (ROC) analysis was performed in order to identify the predictive value of PWV and DRIN for renal outcomes. Cut-points selected were those that yielded the greatest sum of sensitivity and specificity. An explorative subgroup analysis was performed separately in T2DM and EH, in order to ascertain whether the predictive power of biomarkers might be different in the two conditions. A p < 0.05 was considered significant.

## Results

### Clinical characteristics at Visit 0 and 1

Clinical characteristics of the two study groups at Visit 0 are summarized in Table 1. T2DM were significantly older and had a higher BMI as compared to EH; as expected, they showed higher levels of fasting glucose, HbA1c and triglycerides. EH presented higher mean and diastolic BP values. Median UACR, even within the normal range, was higher in T2DM, while eGFR was similar.

**Table 1:** Clinical characteristics of the study groups at visit 0 and visit 1

	EH (n = 43) visit 0	EH (n = 43) visit 1	T2DM (n = 27) visit 0	T2DM (n = 27) visit 1
Age (years)	51.7 ± 8.0	-	60.0 ± 10.5*	-
Men (n,%)	32 (74 %)	-	14 (52 %)	-
Smokers (n,%)	9 (21 %)	-	5 (19 %)	-
BMI (Kg/m^2^)	26.6 (24.6-31.1)	28.4 (25.1-30.1)	30.0 (27.9-36.1)*	29.2 (27.2-35.1)
Brachial systolic BP (mmHg)	142.7 ± 9.9	144.5 ± 17.9	139.8 ± 14.5	141.2 ± 10.7
Brachial diastolic BP (mmHg)	86.1 ± 8.1	86.9 ± 11.8	78.7 ± 8.8*	73.1 ± 9.4
Brachial PP (mmHg)	56.6 ± 8.1	57.6 ± 11.1	61.1 ± 16.6	68.2 ± 13.7
Mean BP (mmHg)	105.7 ± 7.9	107.1 ± 11.5	100.6 ± 7.9*	96.6 ± 7.6
Aortic systolic BP (mmHg)	131.5 ± 11.8	132.4 ± 13.0	128.6 ± 17.2	126.9 ± 10.1
Aortic PP (mmHg)	47.2 ± 13.7	45.2 ± 8.6	51.4 ± 16.4	52.9 ± 13.5
Heart rate (bpm)	65.8 ± 10.5	71.1 ± 10.4^#^	68.5 ± 12.6	68.6 ± 11.8
Fasting plasma glucose (mg/dl)	90 (86–95)	90 (86–96)	140 (119–183)*	114 (107–132)^#^
Hb1Ac (%)	5.2 (5.0-5.7)	5.4 (5.0-6.4)	6.9 (6.4-7.4)*	6.4 (6.1-7.1) ^#^
Total cholesterol (mg/dl)	209 ± 34	206 ± 39	227 ± 53	183 ± 38^#^
LDL-cholesterol (mg/dl)	137 ± 36	127 ± 36	131 ± 57	107 ± 31
HDL-cholesterol (mg/dl)	50 ± 14	53 ± 12	50 ± 17	52 ± 13
Triglycerides (mg/dl)	104 (71–174)	108 (73–162)	164 (102–247)	131 (91–159)
Serum creatinine (mg/dl)	0.90 ± 0.17	0.91 ± 0.18	0.86 ± 0.21	0.85 ± 0.19
eGFR (ml/min/1.73 m^2^)	91.1 ± 13.7	86.9 ± 13.4	86.3 ± 15.1	84.4 ± 14.2
UACR (mg/g)	2.0 (0–5.9)	9.3 (5.7-18.4) ^#^	6.6 (1.6-15.5)*	10.7 (3.8-44.8) ^#^
Nitrotyrosine (μmol/l )	9.8 ± 1.4	12.2 ± 3.2 ^#^	13.1 ± 2.5*	11.9 ± 3.4

*p < 0.05 vs EH group

^#^p < 0.05 vs Visit 0

RI and DRIN values were significantly higher in T2DM as compared to EH (RI 0.66 ± 0.04 vs 0.58 ± 0.04, p < 0.001; DRIN −5.9 ± 5.9 %, −10.1 ± 6.9 %, p = 0.03). Conversely, PWV (8.6 ± 2.1 vs 8.0 ± 1.1 m/s, p = 0.22), AIX (26 ± 15 vs 21 ± 11 %, p = 0.11), BA diameter (4.29 ± 0.63 vs 4.31 ± 0.75 mm, p = 0.91), FMD (3.9 ± 1.5 vs 5.3 ± 2.9 %, p = 0.17) and GTN (5.7 ± 2.6 vs 5.5 ± 2.3 %, p = 0.84) were similar in the two groups.

### Clinical characteristics at Visit 1

Visit 1 took place after a mean period of 4.2 ± 0.6 years from Visit 0 (range 3.4-5.1 years). While at Visit 0 all individuals were untreated, at Visit 1, 25 patients out of 27 in the T2DM group were on pharmacologic glucose-lowering treatment, and 40 patients (13 in the T2DM group and 27 in the EH group) were on BP-lowering drugs. All patients on BP-lowering drugs at Visit 1 were treated with a regimen including a renin-angiotensin system (RAS)-blocker. Office BP during follow-up was unchanged, with a borderline increase in brachial PP in T2DM group (p = 0.07). Glucose control and total cholesterol levels were improved from Visit 0 to Visit 1 in the T2DM subgroup. Serum creatinine and eGFR were substantially unchanged over time, while median UACR was significantly increased. According to the abovementioned definitions, at Visit 1 13 patients (9 T2DM) had developed MA, while 27 patients (11 T2DM) showed eGFR decline.

### Clinical and vascular characteristics according to albuminuria outcome

Incidence of MA was greater in the T2DM than in the EH group (9 vs 4 patients, 33.3 vs 9.3 %, p = 0.005). In the overall population, at baseline, patients developing MA were older (62.2 ± 8.1 vs 53.4 ± 10.4 years, p = 0.03), had higher UACR [15(10–23) vs 2(0–7) mg/g, p = 0.03] and higher nytrotyrosine concentration (13.0 ± 2.7 vs 10.9 ± 2.45 μmol/l , p = 0.048) than those not developing MA. Fasting blood glucose was higher at Visit 0 in patients developing MA [from 172(105–211) to 124(100–141) mg/dl] than in those not [from 95(89–124) mg/dl to 96(90–107) mg/dl], but reached similar values at Visit 1 (p for interaction Visit-Outcome = 0.002). There was no significant difference in RAS blockers use at Visit 1 between patients developing MA or not (78 % vs 53 %, p = 0.17).

**Fig. 1:**
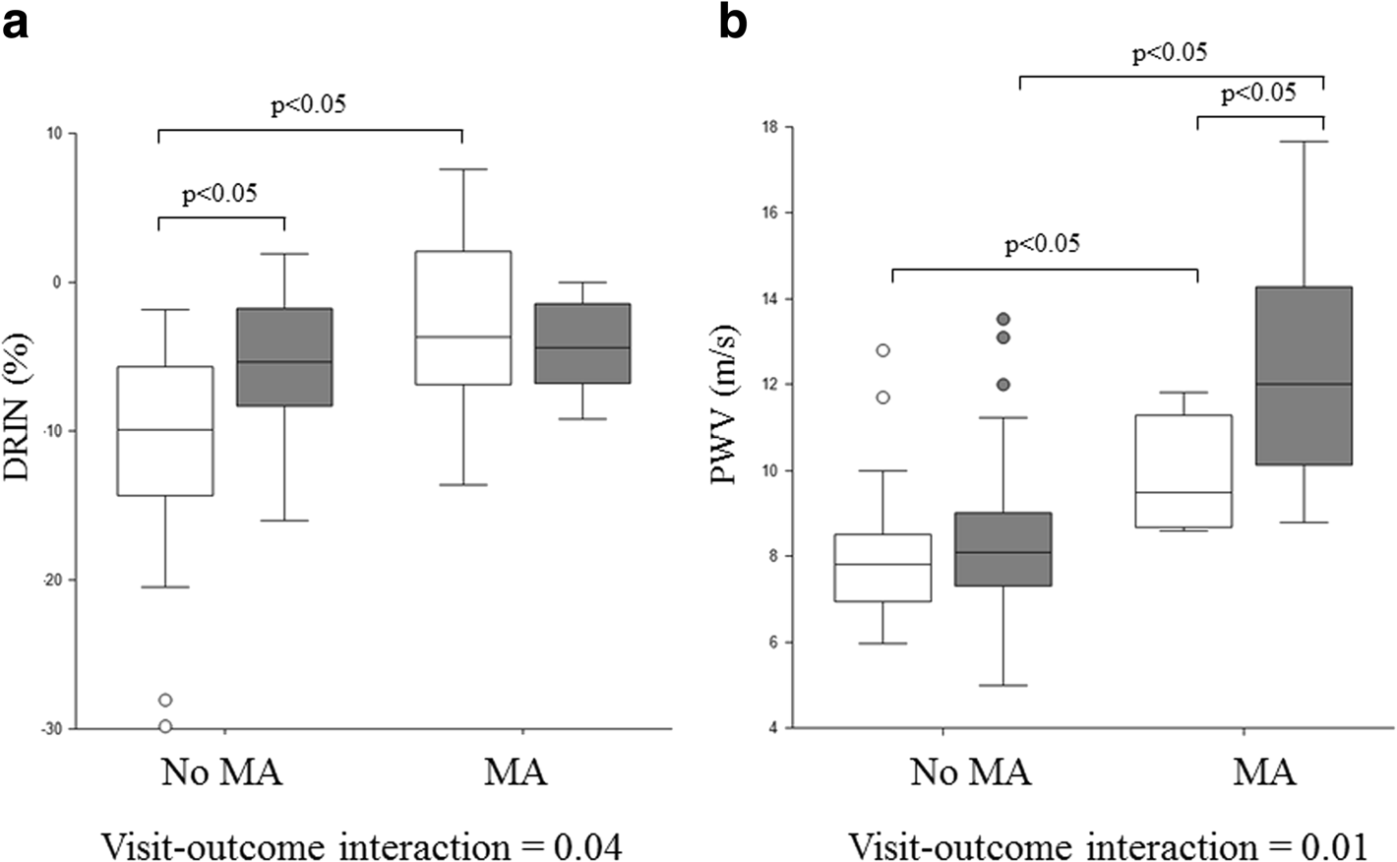
Box plots representing DRIN (**a**) e PWV (**b**) values at Visit 0 (in white) and Visit 1 (in grey) according to microalbuminuria outcome

Baseline vascular characteristics according to MA outcome are shown in Table 2 and Fig. 1. Among all the variables studied, DRIN, PWV and TR were significantly higher in patients developing MA, while RI did not differ significantly. Differences between groups remained significant even after adding age and mean BP as covariates for DRIN and PWV (unadjusted p value: 0.01 and 0.004 respectively; adjusted p value: 0.04 and 0.04 respectively) but not for TR (unadjusted p value 0.03; adjusted p value 0.28). No differences in baseline endothelium-dependent and independent vasodilation of the brachial artery were found in subjects with favorable or unfavorable MA outcome (baseline BA diameter 4.2 ± 0.7 vs 4.7 ± 0.9; FMD 5.1 ± 3.6 vs 3.7 ± 2.3 %; GTN 5.4 ± 3.7 vs 4.2 ± 2.8 %; p = ns for all). In the ROC analysis performed in the overall population, both DRIN greater than −4.55 % or PWV greater than 8.50 m/s were able to predict MA development with a good sensitivity and specificity (Table 3).

**Table 2:** Vascular characteristics according to albuminuria outcome at visit 0 and visit 1 in the whole study group

	Visit 0		Visit 1	
	No MA (n = 57)	MA (n = 13)	No MA (n = 57)	MA (n = 13)
RI	0.60 ± 0.06	0.64 ± 0.05	0.61 ± 0.06	0.67 ± 0.08*
DRIN (%)	−10.6 ± 6.4	−2.8 ± 6.7*	−5.5 ± 4.8#	−4.5 ± 3.1
Aortic SBP (mmHg)	127.0 ± 10.6	134.9 ± 23.0	130.3 ± 12.2	130.2 7.0
Aortic PP (mmHg)	45.4 ± 10.1	52.4 ± 18.8	46.8 ± 11.0	54.9 ± 11.6
AIx (%)	26.7 ± 13.5	30.8 ± 10.0	27.1 ± 10.7	27.8 ± 10.6
TR (ms)	142 ± 19	128 ± 14*	142 ± 13	139 ± 6
Aix@75 (%)	21.6 ± 13.1	26.0 ± 11.0	22.7 ± 9.9	24.1 ± 8.6
PWV (m/s)	7.9 ± 1.5	9.9 ± 1.3*	8.5 ± 1.8	12.3 ± 2.9#

*p < 0.05 vs No MA group

^#^p < 0.05 vs Visit 0

**Table 3:** ROC analysis of vascular variables at visit 0 for MA development in the overall population and in the two subgroups

		AUC	Cut-off	Sensitivity	Specificity
Overall population	DRIN	0.798	−4.55 %	0.84	0.71
	PWV	0.838	8.50 m/s	0.82	1.00
	RI	0.702	0.65	0.82	0.50
T2DM subgroup	DRIN	0.800	−5.16 %	0.83	0.80
	PWV	0.675	9.40 m/s	0.78	0.50
	RI	0.404	0.69	0.92	0.16
HT subgroup	DRIN	0.792	−7.69 %	0.69	1.00
	PWV	0.919	8.60 m/s	0.96	1.00
	RI	0.751	0.63	0.96	0.50

We performed an explorative subgroup analysis in T2DM and EH separately (Table 4). In the T2DM group, baseline DRIN (−8.4 ± 4.1 vs −1.7 ± 7.8 %, p = 0.03) was significantly higher in patients developing MA than in those not. Conversely, in the EH group, baseline PWV (10.5 ± 1.8 vs 7.8 ± 0.8 m/s, p = 0.0004) was significantly higher in patients developing MA than in those not. These data were confirmed by the ROC analysis: in the diabetic sub-group DRIN was a better predictor than PWV, while the opposite occurred in the hypertensive subgroup (Table 3).

**Table 4:** Baseline vascular characteristics according to albuminuria outcome in the two subgroups

	T2DM subgroup		EH subgroup	
	No MA (n = 18)	MA (n = 9)	No MA (n = 39)	MA (n = 4)
RI	0.66 ± 0.04	0.57 ± 0.04	0.57 ± 0.04	0.62 ± 0.05
DRIN (%)	−8.4 ± 4.1	−1.7 ± 7.8*	−11.6 ± 7.0	−5.7 ± 1.6
Aortic SBP (mmHg)	124.7 ± 11.3	128.0 ± 10.3	128.0 ± 10.3	144.0 ± 25.4
Aortic PP (mmHg)	47.5 ± 11.7	44.4 ± 9.3	44.4 ± 9.3	47.0 ± 14.1
AIx (%)	28.3 ± 16.7	25.9 ± 12.0	25.9 ± 12.0	22.5 ± 12.0
TR (ms)	134 ± 25	145 ± 15	145 ± 15	133 ± 30
Aix@75 (%)	24.3 ± 17.3	20.4 ± 10.8	20.4 ± 10.8	18.5 ± 14.8
PWV (m/s)	8.3 ± 2.5	7.8 ± 0.8	7.8 ± 0.8	10.6 ± 1.8*
BA diameter (mm)	4.2 ± 0.4	4.3 ± 0.8	4.3 ± 0.8	4.0 ± 0.3
FMD (%)	4.3 ± 1.3	5.6 ± 4.3	5.6 ± 4.3	4.6 ± 2.9
GTN (%)	6.3 ± 2.6	5.3 ± 3.7	5.3 ± 3.7	4.7 ± 5.4

*p < 0.05 vs No MA group

Then we examined the behavior of vascular characteristics over time according to MA outcome (Table 2). In patients developing MA, DRIN, which was already altered at Visit 0, was unchanged at Visit 1, while in patients not developing MA, a significant increase in DRIN was observed; conversely, PWV showed a further increase only in patients developing MA (Fig. 1).

### Clinical and vascular characteristics according to eGFR outcome

The proportion of patients showing eGFR decline > −1 ml/min/year was similar in EH and T2DM group (16 vs 11 patients, 37.2 % vs 40.7 %, p = ns). In the overall population, patients developing eGFR decline had higher baseline eGFR (93 ± 16 vs 85 ± 11 ml/min1.73, p = 0.04) than those not, while all the other clinical characteristics, including age and nitrotyrosine levels, were similar in the two groups. BP was similar over time in patients developing eGFR decline (mean BP from 103 ± 8 to 103 ± 12 mmHg) and in those not (mean BP from 102 ± 8 to 102 ± 14 mmHg; p for interaction Visit-Outcome = 0.86). The percentage of patients receiving RAS blockers at Visit 1 was similar (62 % vs 56 %, p = 0.66). Fasting blood glucose variation between Visit 0 and 1 was not significantly different in patients developing eGFR decline [from 95(90–135 mg/dl) to 105(91–114)] than in those not [from 96(88–130) to 90(87–110) mg/dl, p for interaction Visit-Outcome =0.53].

Vascular characteristics according to eGFR outcome are shown in Table 5. None of the variables studied was significantly different between those with eGFR slope < −1 ml/min/year during the follow up period and those not. No differences in baseline endothelium-dependent and independent vasodilation of the brachial artery emerged in subjects without or with eGFR decline (baseline BA diameter 4.3 ± 0.8 vs 4.5 ± 0.5 mm; FMD 5.3 ± 4.4 vs 4.0 ± 1.6 %; GTN 5.2 ± 3.7 vs 5.7 ± 2.6 %; p = ns for all).

**Table 5:** Vascular characteristics according to GFR outcome at visit 0 and visit 1 in the whole study group

	Visit 0		Visit 1	
	No GFR decline (n = 43)	GFR decline (n = 27)	No GFR decline (n = 43)	GFR decline (n = 27)
RI	0.61 ± 0.05	0.61 ± 0.06	0.62 ± 0.08	0.61 ± 0.05
DRIN (%)	−9.7 ± 7.9	−6.8 ± 4.5	−5.1 ± 4.6	−5.9 ± 4.3
Aortic SBP (mmHg)	130.2 ± 12.7	129.5 ± 12.1	130.5 ± 13.4	130.4 ± 12.2
Aortic PP (mmHg)	49.5 ± 18.6	47.5 ± 10.2	49.1 ± 13.2	47.0 ± 9.2
AIx (%)	27.3 ± 13.0	27.9 ± 11.8	24.0 ± 9.3	22.4 ± 9.4
TR (ms)	136 ± 20	139 ± 17	138 ± 11	144 ± 12
Aix@75 (%)	23.4 ± 12.1	21.7 ± 12.4	23.4 ± 12.1	21.7 ± 12.4
PWV (m/s)	8.3 ± 1.5	8.1 ± 1.4	8.8 ± 2.0	9.1 ± 2.9

Then we performed subgroup analysis in T2DM and EH separately. In the T2DM group we did not find any significant difference in vascular parameters between patients with different eGFR outcome. Conversely, in EH, DRIN (−5.8 ± 3.5 vs −12.5 ± 7.1 %, p = 0.002) and PWV (8.4 ± 1.1 vs 7.6 ± 0.8 m/s, p = 0.04) were significantly higher in patients whose eGFR declined than in those not, while the other vascular variables were similar (Table 5). Furthermore, in the EH group, nitrotyrosine levels were higher in EH patients with steeper eGFR slope (10.6 ± 1.6 vs 8.9 ± 0.9 μmol/L, p = 0.02), while no difference in nitrotyrosine was found in the overall population and in the T2DM group.

We did not observe any difference in the behavior of vascular characteristics over time according to the slope of eGFR decline (Table 5).

### GTN-induced systemic hemodynamic changes in relation to DRIN

We analyzed brachial and central BP changes after GTN administration, which were available at Visit 0 for 57 out of 70 participants (Table 6), to verify the hypothesis of DRIN dependence on GTN-induced changes in central hemodynamics rather than on renal vasodilation.

**Table 6:** Systemic hemodynamic variables after acute administration of glyceril trinitrate in a subgroup of individuals at Visit 0

Variable	Baseline (n = 57)	Post GTN (n = 57)	P value
Brachial systolic BP (mmHg)	139.8 ± 12.6	134.3 ± 12.1	<0.0001
Brachial diastolic BP (mmHg)	82.3 ± 8.9	80.4 ± 8.6	0.02
Brachial PP (mmHg)	57.5 ± 11.7	53.9 ± 13.2	0.003
Aix (%)	27.4 ± 11.4	23.9 ± 13.0	0.02
Aix@75 (%)	23.7 ± 11.0	19.3 ± 10.8	0.007
Aortic systolic BP (mmHg)	127.6 ± 14.6	122.8 ± 14.2	0.0006
Aortic PP (mmHg)	45.0 ± 12.7	41.5 ± 13.5	0.008
HR (bpm)	67.4 ± 10.6	65.8 ± 9.7	0.08

GTN administration caused a similar reduction in brachial and central PP. AIx was also significantly reduced. GTN-induced absolute change in RI was related to absolute change in central PP (r = 0.39, p = 0.009) and AIx (r = 0.32, p = 0.03), but not brachial PP (r = 0.18, p = 0.20). GTN-induced percent change in RI (that is DRIN) was not related to percent change in brachial or central PP (r = −0.05, p = 0.70 and r = −0.07, p = 0.66 respectively) or AIx (r = −0.20, p = 0.19). Nor absolute (−0.7 ± 13.1 vs −3.3 ± 7.8 mmHg, p = 0.57; 3.9 ± 12.6 vs −5.1 ± 10.0, p = 0.11) neither percent changes in central PP and AIx at Visit 0 (5.1 ± 30.3 vs −7.2 ± 18.6 %, p = 0.36; 19.6 ± 47.5 vs 1.5 ± 134.2 %, p = 0.06) were associated to microalbuminuria development at Visit 1, though a trend for significance was observed for AIx. GTN-induced AIx percent changes at Visit 0 were not significantly different between individuals developing or not microalbuminuria, when age and mean BP were added as covariates (p = 0.50).

## Discussion

CKD, occurring in 25-40 % of T2DM patients [3], accounts for the majority of the diabetes-related morbidity and mortality [26–28]. In hypertensive patients CKD is less frequent [29] but equally deleterious for the global prognosis [30]. For these reasons, the development of a non-invasive test able to predict the onset of any impairment of renal function is appealing. In this pilot, proof-of-concept study we tested the ability of a novel test, DRIN, in predicting microalbuminuria onset and GFR decline in a cohort of newly diagnosed, drug-naive patients with hypertension or T2DM. Furthermore, we compared this novel marker with more established non-invasive biomarkers of renal and systemic vascular damage.

### Predictive role of DRIN for microalbuminuria development

The main finding of this study is that patients developing microalbuminuria after a follow-up of about 4 years had at baseline higher values of PWV and DRIN. Interestingly, the association between these vascular biomarkers and renal outcome seems to be disease-specific: DRIN was the best predictor of microalbuminuria onset in T2DM patients, PWV in hypertensive patients.

It has been demonstrated that high resting RI (>0.80) predicts rapid eGFR decline in long-standing microalbuminuric hypertensive T2DM patients [31]. This is not necessarily in contrast with the results of our study, enrolling only newly diagnosed T2DM patients, all with normal albumin excretion and normal RI at baseline. In fact, we demonstrated that renal vasodilating capacity is reduced in T2DM patients before the onset of established renal damage and in the presence of normal RI values [20]. In this early phase, it is conceivable that functional, rather than structural alterations might be already present, indicating a subclinical stage of renal damage. Several hypotheses on the pathogenesis of microalbuminuria in T2DM have been suggested, many of them involving alterations of the glomerular endothelium [32, 33]. DRIN is a simple vascular reactivity test, exploring the renal district, which might unmask subclinical vascular alterations possibly responsible for albuminuria development. There has been a recent large debate on the clinical significance of RI, with new evidence indicating that this index might reflect systemic hemodynamic status and arterial impedance rather than a truly “renal” condition [18]. Following this hypothesis, the correlation between RI and microalbuminuria found in some studies may reflect the effect of increased pulsatility in the aorta, leading to microvascular damage over time [19]. Nitrates are known to have a significantly greater impact on hemodynamic pulsatile load, and in particular on wave reflection [8], thus it is mandatory to determine whether DRIN reflects a systemic rather than a renal condition, as suggested for RI. Our data support the hypothesis that GTN-induced changes in central hemodynamics (represented by central PP and AIx) are major determinants of DRIN, but do not retain any predictive value for renal outcomes, suggesting a renal-specific significance for DRIN. Furthermore, in the present study we demonstrated that, in comparison to PWV, DRIN retains a greater predictive value for microalbuminuria onset in T2DM, suggesting that RI changes after nitrate administration may overcome the shortcomings highlighted for RI.

### DRIN and PWV changes during follow-up

In this study we explored the possible clinical significance of variations of vascular biomarkers over time. Conceivably DRIN may play a role in prediction of incipient nephropathy, particularly in T2DM, while its ability to serve as a marker of damage accrual is less likely, since it is already severely altered in newly diagnosed patients and it cannot show further deterioration during follow-up. Thus, we might speculate that the predictive value of DRIN is present only at the very beginning of the diabetic and hypertensive disease, since DRIN rapidly deteriorates over time in all individuals regardless of renal prognosis. DRIN, being an early marker of renal involvement, probably loses its usefulness with disease progression. On the other hand, PWV not only is early increased, but also progresses faster in patients developing microalbuminuria, proposing arterial stiffness as both a cause and a consequence of renal damage accrual, particularly in hypertensive individuals. In the hypertensive subgroup PWV, but not DRIN, was higher at baseline in those developing microalbuminuria, confirming previous cross-sectional observations [34, 35] and suggesting that mechanisms responsible for its onset are different for hypertension or type 2 diabetes [36]. In particular, hypertension-related large artery stiffness, reducing the buffering capacity of the arterial tree, exposes peripheral circulation and small vessels to increased flow and pressure pulsatility, thus inducing microvascular damage [37].

### Predictive role of DRIN for eGFR decline

Vascular biomarkers were not able to predict GFR decline neither in the overall study population nor in the T2DM subgroup. This finding might be due to the slow rate of GFR progression in our population, which was modest and similar in the hypertensive and T2DM groups, in agreement with the short disease duration and the fairly good metabolic and blood pressure control. However the apparent discrepancy between the demonstrated predictive role of vascular biomarkers for MA onset but not for eGFR outcome in diabetic individuals is not surprising, since recent evidence suggest that MA onset and eGFR decline often do not coexist [38]. Thus we may speculate that specific pathophysiological mechanisms are involved in the development of the emerging phenotype of normoalbuminuric eGFR decline [38]. It is also interesting to note that the only predictor of eGFR decline was a higher eGFR at baseline, in agreement with the hypothesis that hyperfiltration may contribute to diabetic nephropathy onset [39].

DRIN and PWV, as well as nitrotyrosine, are early compromised in EH patients with steeper eGFR slope, suggesting that among mechanisms of renal damage development in hypertension, local and systemic vascular alterations, together with increased oxidative stress, may play a crucial role. It is well known from experimental studies that renal oxidative stress contributes to renal vasoconstriction and ischemia, thus favoring hypertension development and renal function decline [40]. Noteworthy, we excluded major systemic hemodynamic variations as a determinant of eGFR decline in hypertensive patients, since BP behavior over time was similar in patients experiencing eGFR decline and in those not, and a similar proportion of patients had started a treatment with BP-lowering drugs during follow-up in the two groups with different outcomes. If our observations will be confirmed in larger cohorts of individuals, DRIN might be potentially useful for the early identification of hypertensive patients at risk of developing renal impairment.

### Limitations

We must acknowledge as limitations of this study the relatively low sample size, which did not allow us to verify the independent predictive value of DRIN and to fully assess its clinical significance in the diabetic and hypertensive subgroup. In particular, given the small size of the T2DM group, it was not possible to perform a multivariate analysis and demonstrate whether the predictive role of DRIN for microalbuminuria onset is independent of other well-known determinants such as glycemic control and baseline UACR levels. Further prospective, adequately powered studies, should test the additive predictive value of DRIN on top of these clinical characteristics, validating this test to be introduced in the clinical practice. Finally, the use of estimated GFR instead of its direct measurement might have made less accurate results regarding renal function decline.

## Conclusions

This proof-of-concept, prospective study demonstrates for the first time that renal vasodilating capacity is early reduced in patients with T2DM and EH at risk of developing incipient nephropathy. DRIN appears to be superior to systemic vascular biomarkers such as PWV for prediction of microalbuminuria onset, when measured in newly diagnosed, untreated patients with T2DM, since it probably retains a clinical significance that is specific for the kidney. Moreover, this study highlights the importance of local vascular mechanisms in the pathogenesis of CKD in both EH and T2DM. Larger prospective studies are needed to ascertain whether DRIN has an additive predictive value in comparison to clinical predictors of CKD, as well as its usefulness in the clinical setting.

## Abbreviations

AIx: Augmentation index
BP: Blood pressure
CKD: Chronic kidney disease
DRIN: Dynamic renal resistive index
EH: Essential hypertensive patients
FMD: Flow mediated dilation
eGFR: Estimated glomerular filtration rate
GTN: Glyceryl trinitrate
MA: Microalbuminuria
PP: Pulse pressure
PWV: Pulse wave velocity
RAS: Renin-angiotensin system
RI: Renal resistive index
T2DM: Type 2 diabetic patients
TR: Time to reflection
UACR: Urine albumin to creatinine ratio
